# Real world scenarios in rare variant association analysis: the impact of imbalance and sample size on the power in silico

**DOI:** 10.1186/s12859-018-2591-6

**Published:** 2019-01-22

**Authors:** Xinyuan Zhang, Anna O. Basile, Sarah A. Pendergrass, Marylyn D. Ritchie

**Affiliations:** 1 Genomics and Computational Biology Graduate Group, Perelman School of Medicine, University of Pennsylvania, Philadelphia, PA USA; 2 Department of Biomedical Informatics, Columbia University, New York, NY USA; 3 Biomedical and Translational Informatics Institute, Geisinger, Danville, PA USA; 4 Department of Genetics, University of Pennsylvania, Perelman School of Medicine, Philadelphia, PA USA

**Keywords:** Rare variant association analysis, Sample size imbalance, Power analysis, Simulation study

## Abstract

**Background:**

The development of sequencing techniques and statistical methods provides great opportunities for identifying the impact of rare genetic variation on complex traits. However, there is a lack of knowledge on the impact of sample size, case numbers, the balance of cases vs controls for both burden and dispersion based rare variant association methods. For example, Phenome-Wide Association Studies may have a wide range of case and control sample sizes across hundreds of diagnoses and traits, and with the application of statistical methods to rare variants, it is important to understand the strengths and limitations of the analyses.

**Results:**

We conducted a large-scale simulation of randomly selected low-frequency protein-coding regions using twelve different balanced samples with an equal number of cases and controls as well as twenty-one unbalanced sample scenarios. We further explored statistical performance of different minor allele frequency thresholds and a range of genetic effect sizes. Our simulation results demonstrate that using an unbalanced study design has an overall higher type I error rate for both burden and dispersion tests compared with a balanced study design. Regression has an overall higher type I error with balanced cases and controls, while SKAT has higher type I error for unbalanced case-control scenarios. We also found that both type I error and power were driven by the number of cases in addition to the case to control ratio under large control group scenarios. Based on our power simulations, we observed that a SKAT analysis with case numbers larger than 200 for unbalanced case-control models yielded over 90% power with relatively well controlled type I error. To achieve similar power in regression, over 500 cases are needed. Moreover, SKAT showed higher power to detect associations in unbalanced case-control scenarios than regression.

**Conclusions:**

Our results provide important insights into rare variant association study designs by providing a landscape of type I error and statistical power for a wide range of sample sizes. These results can serve as a benchmark for making decisions about study design for rare variant analyses.

**Electronic supplementary material:**

The online version of this article (10.1186/s12859-018-2591-6) contains supplementary material, which is available to authorized users.

## Background

During the last decade, Genome-Wide Association Studies (GWAS) have greatly advanced our understanding of the impact of common variants on complex traits. The associations of alleles with frequency more than 1–5% have provided important insights into research and clinical practice [1, 2]. Despite GWAS revealing novel disease associations, limited genetic heritability has been explained by GWAS results [3]. Rare alleles, with moderately large genetic effect sizes, may explain more of the phenotypic variance of complex disease [4]. Low frequency or rare variants may have an essential contribution to unexplained missing heritability [5, 6]. The development of sequencing technologies has increased access to rare variation data for large sample sizes. However, it is crucial to better understand the statistical power and analytic limitations of rare variant association approaches.

Due to the low frequency of rare variants, single locus association tests in traditional GWAS are underpowered for rare variant association analysis [7] unless the casual variants have very large effect sizes [8]. To boost power, region-based collapsing or binning approaches have become a standard for analyzing rare variants [7]. These methods evaluate the association of the joint effect of multiple rare variants in a biologically relevant region with the outcome [8].

Numerous association methods have been developed [7, 9–18], and this manuscript focuses on evaluating two of the most commonly used approaches for gene-based testing, burden and dispersion, using a simulation approach. Burden tests summarize the cumulative effect of multiple rare variants into a single genetic score and test the association between this score and phenotypic groups using regression [8]. The major assumption of burden tests is that all rare variants in a group have the same direction and magnitude of effect on the trait [8], and violation of this assumption leads to a loss of power [14]. Dispersion tests, on the other hand, evaluate the distribution of genetic effects between cases and controls by applying a score-based variance-component test [8]. The sequence kernel association test (SKAT) is a widely used dispersion method. It applies a multiple regression model to directly regress the phenotype on genetic variants in a region, followed by a kernel association test on the regression coefficients [9]. SKAT is robust to the magnitude and direction of genetic effects as well as to the presence of neutral variants, or a small portion of disease variants [8, 9].

Statistical power for both burden and dispersion tests has been assessed in many simulation settings [7, 9, 15, 19, 20], however, these simulations have focused on an equal (or balanced) number of cases and controls. In real data scenarios, researchers often have unequal (or unbalanced) number of cases and controls. With the application of association methods on unbalanced samples, it is beneficial to acquire the expected type I error and power to guide the study design for rare variant association tests. For example, for diseases that have a low prevalence in the population, what number of cases and how many controls are necessary to detect the impact of rare variation on the disease? In Phenome-Wide Association Studies (PheWAS) [21] there are potentially a wide range of case and control numbers and overall sample sizes across hundreds of diagnoses and traits [22–24]. A challenge for PheWAS studies using rare variants is to understand the impact of varying sample sizes, varying case numbers, and genetic effect sizes [24].

In this study, we performed extensive simulation analyses to assess the influence of sample size on the type I error and power distribution for regression (a burden test) and SKAT (a dispersion test). We designed twelve balanced sample size datasets and twenty-one unbalanced sample size scenarios. Since a large sample size has been widely known as a necessity for detecting significant rare variant associations [7, 8, 25], in this paper, we mainly simulate unbalanced scenarios using a large total sample size. BioBin [26–30] was used for rare variant binning and association testing. Results on the statistical performance of both logistic regression and SKAT can serve as a benchmark for making decisions about future rare variant association studies.

## Results

We evaluated burden-based tests using logistic regression and dispersion-based tests using SKAT. All associations are evaluated for a binary outcome on a simulated gene with an average of 143 rare variant loci. We varied the number of cases, controls, and also the balance between cases and controls. All reported results here have a MAF upper bound (UB) set at 0.01. The supplementary material (Additional file 1: Figures S1 and S2) shows results with a MAF upper bound (UB) of 0.05.

### Type I error results

**Fig. 1:**
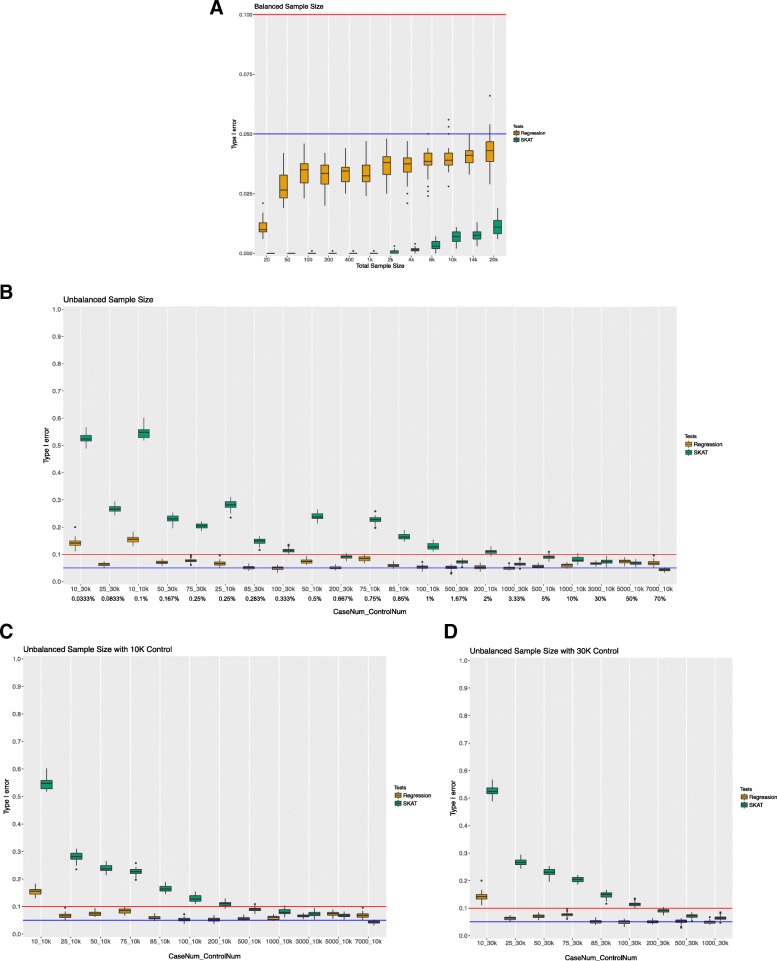
Type I error simulation results with MAF UB of 0.01. For visualization and comparison purposes, blue and red horizontal lines indicate type I error at 0.05 and 0.1 respectively. Fig. (**a**) shows the results for type I error for an equal number of cases and controls for differing sample sizes. Note that the y-axis only goes to a type I error rate of 0.1. Fig. (**b**) shows the type I error rate for different unbalanced cases and controls as arranged by case to control ratio. The axis is labeled by the number of cases then the number of controls for each simulation. The percentage of cases to controls is also listed below the number of cases and controls. Figs. (**c** and **d**) show the results as ordered by the number of cases. Figure 1**c** has 10,000 control and Fig. 1**d** has 30,000 control

Figure 1 displays the overall type I error simulation results for both balanced and unbalanced sample sizes. As shown in Fig. 1a, with balanced number of cases and controls, the type I error for both regression and SKAT is well controlled under 0.05 with a few exceptions (the type I error for these was still below 0.1). Interestingly, regression had an overall higher type I error rate compared with SKAT for balanced samples. In addition, SKAT had an overall slightly increased type I error as the overall sample size increased. For regression, however, with increasing overall sample size, we did not observe an overall increasing trend in the Type I error rate. Similar results have also been observed with MAF UB of 0.05 (Additional file 1: Figure S1A).

For unbalanced sample sizes, we investigated whether the type I error rate was driven by the ratio of the cases to controls or by the number of cases when having a large control sample. We ordered the sample sizes by case to control ratio in Fig. 1b, and by case number within the same control sample size in Fig. 1c and Fig. 1d. The type I error distribution for differing numbers of cases regardless of the number of controls had similar trends (Fig. 1c and Fig. 1d). Thus, our results suggest that number of cases tends to drive the type I error rate in addition to the case to control ratio under large control group scenarios.

An overall higher type I error rate in unbalanced case-control ratios (Fig. 1b) was observed compared to balanced case-control ratios (Fig. 1a) for both tests, most of which are higher than 0.05. Contrary to what was seen in balanced samples, type I error rates for SKAT were overall higher than regression. An exception to this for SKAT is seen when the case number increase significantly such as 5000 and 7000 cases with 10,000 controls. Overall, for SKAT there is decreasing type I error trend as case number increases (Fig. 1c and Fig. 1d). Regression, on the other hand, has a relatively consistent type I error in the unbalanced case control ratio tests.

### Power results

#### Odds ratio 2.5

**Fig. 2:**
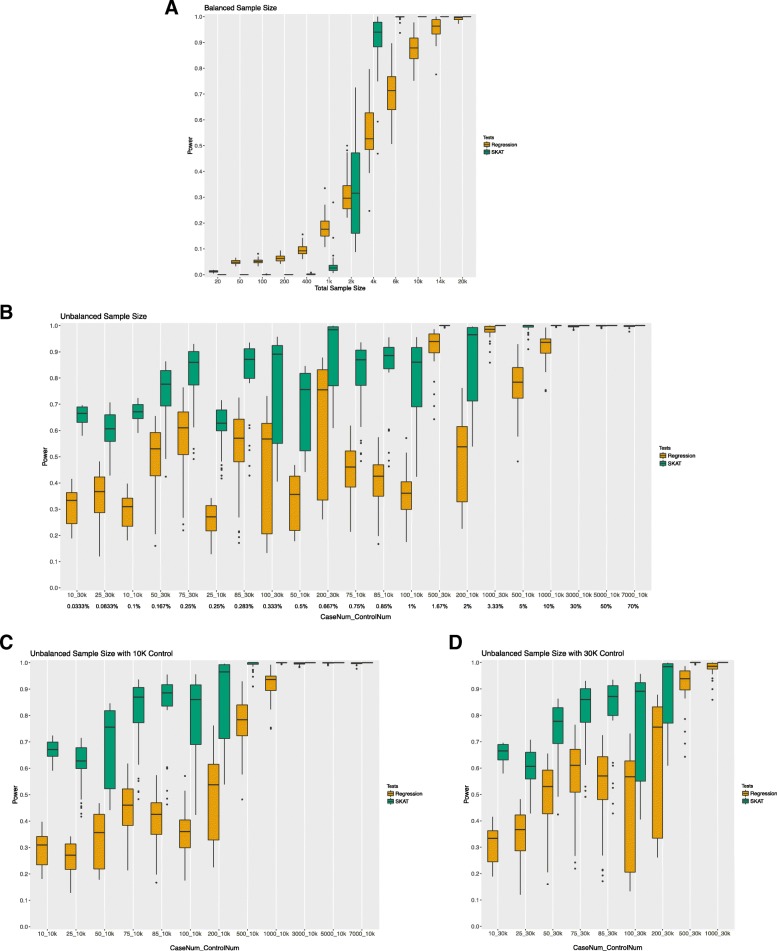
Power simulation results with cutoff for evaluated variation of MAF 0.01. Fig. (**a**) shows the results when cases and controls are equal in number. Fig. (**b**) shows the impact of unbalanced cases and controls on power ranked by the case/control ratio. The percent case to control ratio is listed below the x-axis. Figs. (**c** and **d**) show the results for power with unbalanced cases and controls ordered by case number with 10,000 controls (**c**) and 30,000 controls (**d**)

For balanced numbers of cases and controls and an odds ratio 2.5 for rare disease loci, the power distribution is shown in Fig. 2a. The results indicate that regression has relatively higher power than SKAT for a sample size less than 1000, while SKAT has higher power given larger sample sizes (≥4000). For a total sample size less than 2000, both methods have less than 50% power to detect true positive effects. In order to achieve 90% power, a total balanced sample size of 4000 is needed for SKAT and nearly 14,000 is needed for regression, based on our power simulation settings.

Importantly, SKAT has an overall higher power for unbalanced cases and controls than regression (Fig. 2b). Similar to the type I error distribution, power was also driven by the number of cases instead of the ratio of cases to controls under large control group scenarios (Fig. 2b-d). Notably, overall power was improved whether tested via SKAT or regression approach with an unbalanced case control ratio compared to the balanced case control ratio simulations.

The power analyses for unbalanced samples suggest an overall increasing trend as the number of cases increases. Based on the MAF UB of 0.01 results (Fig. 2c and d), SKAT power for an unbalanced number of cases with case numbers larger than 200 does yield a mean power over 90%. For regression with an unbalanced sample size, more than 1000 cases would yield a mean power of 90% under a 10,000 controls sample size, while case numbers more than 500 would yield the same power under a 30,000 subject control sample size. The same trend has been observed for a MAF UB of 0.05 (Additional file 1: Figure S2c and d).

#### Mixture of genetic variation contributing to risk and protection for outcome

The above power simulations were performed on 10 disease loci where rare variants had an odds ratio 2.5 contributing to risk. In order to better assess the performance of statistical methods, we designed three sets of models containing variants contributing to both protection and risk with varied effect sizes for 10 disease loci (see Methods for more details). We compare four scenarios here: an upper bound on simulated rare variants with a MAF of 0.01 and 0.05; a balanced sample size with 2000 cases and 2000 controls, and an unbalanced sample size with 200 cases and 10,000 controls. We chose these sample sizes from the results of our previous simulations as we observed both regression and SKAT to have adequate power and controlled type I error with these case control numbers.

**Fig. 3:**
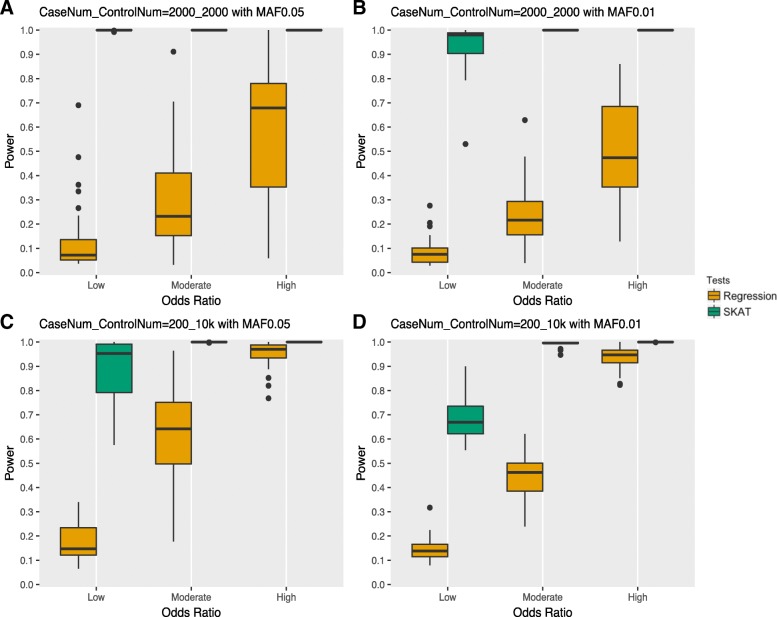
Power comparison of three models with differing contributions from protective and risk rare genetic variation. The results are shown for variants contributing low, moderate, or high impact on outcome risk or protection. Methods describe the range of odds ratios corresponding to the different categories. (**a**) Total sample size of 4000 for balanced cases and controls with MAF UB 0.05. (**b**) Total sample size of 4000 for balanced cases and controls with MAF UB 0.01. (**c**) 200 cases and 10,000 controls with MAF UB 0.05. (**d**) 200 cases and 10,000 controls with MAF UB 0.01

As shown in Fig. 3, the power increases as the impact of rare variation on outcome increases. SKAT outperforms regression in all scenarios, which is expected since the power for burden tests decrease when both protective and risk effects are present. Comparing a MAF UB of 0.05 (left two plots) and a MAF UB of 0.01(right two plots) indicates that SKAT has higher power for MAF UB of 0.05 whereas regression doesn’t have distinguishable power differences. When comparing the top two plots of Fig. 3 with the bottom two plots, we observe higher power for regression in unbalanced samples with 200 cases and 10 k controls compared to 2000 cases and 2000 controls. However, the opposite trend was observed for SKAT.

## Discussion

Previous simulation studies have been conducted to characterize the statistical performance for burden and dispersion-based approaches using a balanced population of cases and controls [7, 9, 19, 20]. However, there are many scenarios where there may not be balanced case control data for a study, and it is important to know if this will be impactful as rare variant association methods evaluate the joint effect of multiple rare variants between case and control groups. In this study, we sought to evaluate the influence of case control balance on the statistical performance of logistic regression and SKAT rare variant methods.

We found an overall higher type I error rate for unbalanced samples (mostly above 0.05) compared with balanced samples (mostly below 0.05) for both tests, suggesting that an unequal number of cases and controls has a clear statistical impact on type I error for rare variant association analysis. Previous research has reported that the type I error rate for SKAT is conservative for smaller sample sizes [9]. Indeed, our balanced sample size simulations suggest the same trend. However, SKAT has an inflated type I error for unbalanced samples with cases less than 200, thus we recommend researchers interpret those results with caution. Interestingly, regression shows a well-controlled type I error rate for both balanced and unbalanced samples. If controlling type I error is the priority, logistic regression is a more appropriate method than SKAT for both balanced and unbalanced scenarios.

Statistical power largely depends on the number of disease loci and the odds ratio. In this paper, we evaluated both same-direction signal (i.e. 2.5 odds ratio) and mixed odds ratio models (Table 3) on 10 disease loci out of an average of 143 rare variant loci. We assessed the power distribution across various sample sizes using an odds ratio of 2.5. For balanced samples, given that both SKAT and regression have an overall controlled type I error, a total sample size less than 2000 obtains power less than 50% and more than 4000 obtains power higher than 50%. For unbalanced sample scenarios, SKAT has an overall higher power distribution than regression. Results show that at least 200 case samples are needed to obtain a power of 90% via SKAT, and an even larger number of cases are required for the regression approach.

As for models with a range of variants contributing to risk and protection for an outcome, our results suggest that SKAT has an overall higher power compared with logistic regression. The results are expected since burden tests lose power when variants contribute to a range of risk and protection for an outcome. Understandably, as the impact of the rare variants on outcome increases, power increases for all scenarios.

Based on our type I error and power results across various unbalanced sample sizes, a clear trend exists between these statistics and the number of cases in addition to the case to control ratio (simulation results of constant case to control ratio are shown in Additional file 1: Figure S3). As many studies ensure the proper case to control ratio, we also recommend that researchers pay attention to the number of cases in the rare variation association studies to help achieve expected type I error and power rates. To our knowledge, our work is the first to propose the landscape of statistics while varying the balance of sample sizes for rare variant association methods.

The likely reason that our simulations present relatively lower power for regression could be a small proportion of disease loci being simulated. As the number of disease loci increases, we expect to observe higher power for burden-based approaches. Future work will aim to simulate various disease loci and odds ratio combinations to provide comprehensive implications on power assessment.

## Conclusion

In this paper, we have presented a simulation study through a wide range of balanced and unbalanced sample sizes, to fully assess the type I error and power distribution for burden and dispersion based rare variant association methods. We observe an impact of sample size imbalance on the statistical performance which can serve as a benchmark for future rare variant analysis.

## Methods

### BioBin

BioBin is a C++ command line tool that performs rare variant binning and association testing via a biological knowledge driven multi-level approach [29]. The framework of a BioBin analysis is to group rare variants into “bins” based on user-defined biological features followed by statistical tests upon each bin. Biological features, which include genes, inter-genic regions, pathways, and others, are defined by prior knowledge obtained from the Library of Knowledge Integration (LOKI) database [26]. LOKI is a local repository which unifies resources from over thirteen public databases, such as the National Center for Biotechnology dbSNP and gene Entrez database information [31], Kyoto Encyclopedia of Genes and Genomes [32], Pharmacogenomics Knowledge Base [33], Gene Ontology [34], and others. Several select burden and dispersion-based statistical tests have been implemented into BioBin [27, 29], namely linear regression, logistic regression, Wilcoxon rank-sum test, and SKAT [9], which allows users the option of choosing the appropriate test(s). All of the statistical tests have been retained as their original statistical testing framework within BioBin. BioBin also enables users to perform association analysis across multiple phenotypes in a rare variant PheWAS. In this paper, we evaluate power and type I error using both logistic regression and SKAT using the BioBin 2.3.0 software [29]. BioBin software and the user manual are freely available at Ritchie Lab website [35] .

### Simulation design

#### Sample size and case control ratios

Simulations were designed to systematically evaluate the impact of different sample sizes, as well as different case control ratios for rare variant association tests. Twelve different scenarios for a balanced number of cases and controls with a total sample size ranging from 20 to 20,000 were simulated. For unbalanced scenarios, a wide range of tests were constructed with case numbers varying from 10 to 7000 and two sets of large control samples (10,000 and 30,000). Case to control ratio was calculated as the number of cases divided by the number of controls. Details of the study design with respect to sample size are shown in Table 1. Moreover, we also designed a few simulations with larger control group (50,000; 100,000; and 200,000), results of which are shown in the Additional file 1: Table S1. Finally, it is important to note that the results would be comparable even if the scenario is reversed and the data include more cases than controls. As long as the customized Madsen and Browning weighting scheme is used, then the results would be the same whether the data include 1000 cases and 100 controls or 100 cases and 1000 controls (Additional file 1: Figure S4).

**Table 1:** Simulation Design

Balanced Cases and Controls	
Total Sample Size 20, 50, 100, 200, 400, 1000, 2000, 4000, 6000, 10,000, 14,000, 20,000	
Unbalanced Cases and Controls	
Number of controls 10,000	Number of controls 30,000
Number of cases 10, 25, 50, 75, 85, 100, 200, 500, 1000, 3000, 5000, 7000	Number of cases 10, 25, 50, 75, 85, 100, 200, 500, 1000

#### Minor allele frequency

Minor allele frequencies (MAFs) were randomly assigned to our simulated rare variants using allele frequency distribution data from actual whole exome sequencing data from 50,726 patients from the MyCode Community Health Initiative as a part of the DiscovEHR project [36]. Due to the rounding precision of MAF that SeqSIMLA2 [37] requires, we used 0.0015 as the MAF lower boundary to avoid zero MAF for simulated variants. For the MAF upper bound (MAF UB), we simulated two sets of data, one with MAF UB 0.01 and the other with MAF UB 0.05, respectively.

#### Parameter settings

As our primary goal is to compare the effect of case-control sample sizes, we set other parameters as constant across all the datasets (Table 2). All simulations were generated with an average of 143 loci per dataset as we calculated this to be the mean number of rare loci from 800 genes in a recent PheWAS study [38]. Here, “locus” refers to a genetic location which harbors genetic variants. We also applied a customized Madsen and Browning [12] weighting scheme as implemented in BioBin for all datasets in order to increase statistical power [27].

**Table 2:** Other Parameter Settings

Number of Simulations	1000 runs times 30 replicates for each sample size scenario
Upper Threshold for MAF	0.01 and 0.05
Variant Weighting	Madsen and Browning [12]
Disease Prevalence	5%
Number of Disease Loci	10
Odds Ratio (OR)	All disease loci with OR 2.5; Half of disease loci with risk effect, the other half with protective effect

#### Simulation model

All of the datasets were generated using the software SeqSIMLA2.8, which can be used to design simulated datasets given user-specified sample size, effect sizes for genetic traits, and genetic model [37]. The disease penetrance model in SeqSIMLA is based on a logistic function [37]:

$$ \mathrm{logit}\ \left(\mathrm{P}\left(\mathrm{case}\right)\right)=\upalpha +{\upbeta}_1{\mathrm{x}}_1+{\upbeta}_2{\mathrm{x}}_2+{\upbeta}_3{\mathrm{x}}_3+\dots +{\upbeta}_{\mathrm{p}}{\mathrm{x}}_{\mathrm{p}} $$

x_1_, x_2_, x_3_, …, x_p_ represent the genotypes across p disease loci. β_1_, β_2_, β_3_, …, β_p_ represent the log of the odds ratios. SeqSIMLA will search for α so that the disease prevalence is close to the specified prevalence. Here, disease prevalence was set to 5%.

#### Type I error (T1E) and power simulation

Each type I error or power value was calculated from 1000 independent simulated datasets with significance assessed at α = 0.05. We replicated 1000 runs 30 times as to account for sampling variability. Running 30 replicates of 1000 datasets was optimal to reduce computational and memory burden. The simulated data did not have any missingness in either genotype or phenotype. Type I error was obtained from null datasets with no genetic association signal. For power, 10 random disease loci with an odds ratio of 2.5 per locus were simulated. In our study, power is defined as the probability of detecting a true signal (i.e. to reject the null hypothesis) when the null hypothesis is false. Power is calculated as the number of datasets that have rejected the null hypothesis at α = 0.05 level divided by the total number of datasets (i.e. 1000). We also designed three sets of mixed odds ratio models where half of the 10 disease loci had protective effects, and half had risk effects, as described more in the next section.

#### Mixed odds ratio models

For most of the simulations, an odds ratio of 2.5 was used for 10 disease loci, indicating consistent risk for all associated rare variants. We also designed three types of protective and risk odds ratio combinations for the 10 disease loci. The detailed odds ratio for 10 disease loci are shown in Table 3, where variants were assigned a range of “Low”, “Moderate”, or “High” risk or protective impact, randomly. For each mixed model, we calculated protective (OR < 1) effect as the same as the risk effect as to retain the consistent range of association signals.

**Table 3:** Detailed Parameters for Mixture Odds Ratio Design

	Randomly Selected 10 Disease loci									
Signal Level	OR > 1 range (Risk)					OR < 1 range (Protective)				
Low	**2.3**	2.73	3.15	3.58	**4**	0.43	0.37	0.32	0.28	0.25
Moderate	**4**	5.25	6.5	7.75	**9**	0.25	0.19	0.15	0.13	0.11
High	**9**	11.5	14	16.4	**19**	0.11	0.087	0.07	0.06	0.053

Note: The numbers in bold represent the boundaries when selecting the odds ratios

#### Boxplot

All of the boxplots were generated using the “geom_boxplot” function within “ggplot2” R package [39]. The “reshape2” R package [40] was used for format changing purposes. Each boxplot bar represents the distribution of type I error or power calculated from 30 replicates.

## Additional files

Additional file 1: **Figure S1** and **S2.** Type I error and power simulation results with MAF upper bound of 0.05. **Figure S3.** Type I error and power simulation results using a constant case to control ratio. **Figure S4.** Type I error comparison when case control sample size is reversed. **Table S1.** Simulation results for case sample size of 200 and control sample size of 50 k, 100 k and 200 k. (PDF 551 kb)

Additional file 2: A summary of results for type I error and power simulations with MAF upper bound of 0.01. (XLS 109 kb)

## Abbreviations

GWAS: Genome-Wide Association Studies
MAF UB: Minor allele frequency upper bound
MAFs: Minor allele frequencies
OR: Odds Ratio
PheWAS: Phenome-Wide Association Studies
SKAT: Sequence Kernel Association Test
