# Supplementary material for: Real world scenarios in rare variant association analysis: the impact of imbalance and sample size on the power in silico
Source: BMC Bioinformatics. 2019 Jan 22;20:46. doi: 10.1186/s12859-018-2591-6 (PMC6343276; doi:10.1186/s12859-018-2591-6)
Supplement: Supplementary file 1 — Figure S1 and S2. Type I error and power simulation results with MAF upper bound of 0.05. Figure S3. Type I error and power simulation results using a constant case to control ratio. Figure S4. Type I error comparison when case control sample size is reversed. Table S1. Simulation results for case sample size of 200 and control sample size of 50 k, 100 k and 200 k. (PDF 551 kb) [file 12859_2018_2591_MOESM1_ESM.pdf]

Real world scenarios in rare variant association analysis: the impact of imbalance and sample size on the power *in silico*

Supplementary material

Xinyuan Zhang[1], Anna O. Basile[2], Sarah A. Pendergrass[3], Marylyn D. Ritchie[1,4]

1. Genomics and Computational Biology Graduate Group, Perelman School of Medicine, University of Pennsylvania, Philadelphia, PA
2. Department of Biomedical Informatics, Columbia University, New York, NY
3. Biomedical and Translational Informatics Institute, Geisinger, Danville, PA
4. Department of Genetics, University of Pennsylvania, Perelman School of Medicine, Philadelphia, PA

December 18, 2018

Contents

1. Type I error simulation results with MAF UB of 0.05 1

2. Power simulation results with with MAF UB of 0.05 3

3. Type I error and power simulation results using a constant ratio with MAF UB of 0.01 4

4. Type I error comparison when case control sample size is reversed 5

5. Simulation results for case sample size of 200 and control sample size of 50k, 100k and 200k with MAF UB of 0.01 5

1. Type I error simulation results with MAF UB of 0.05

A

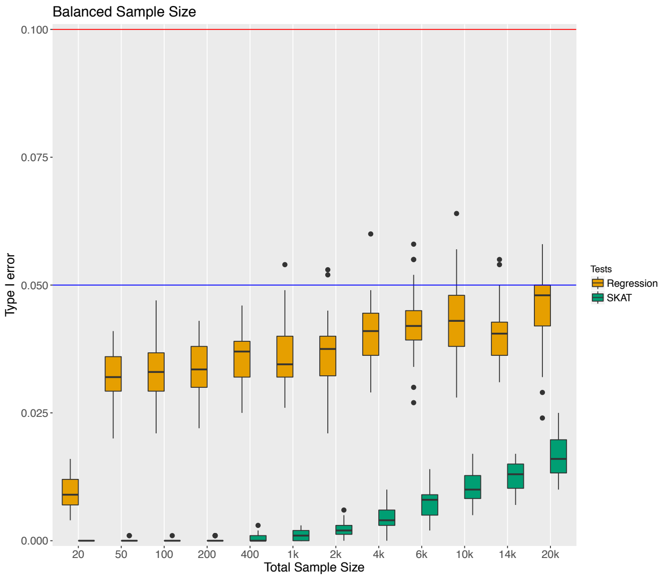

B

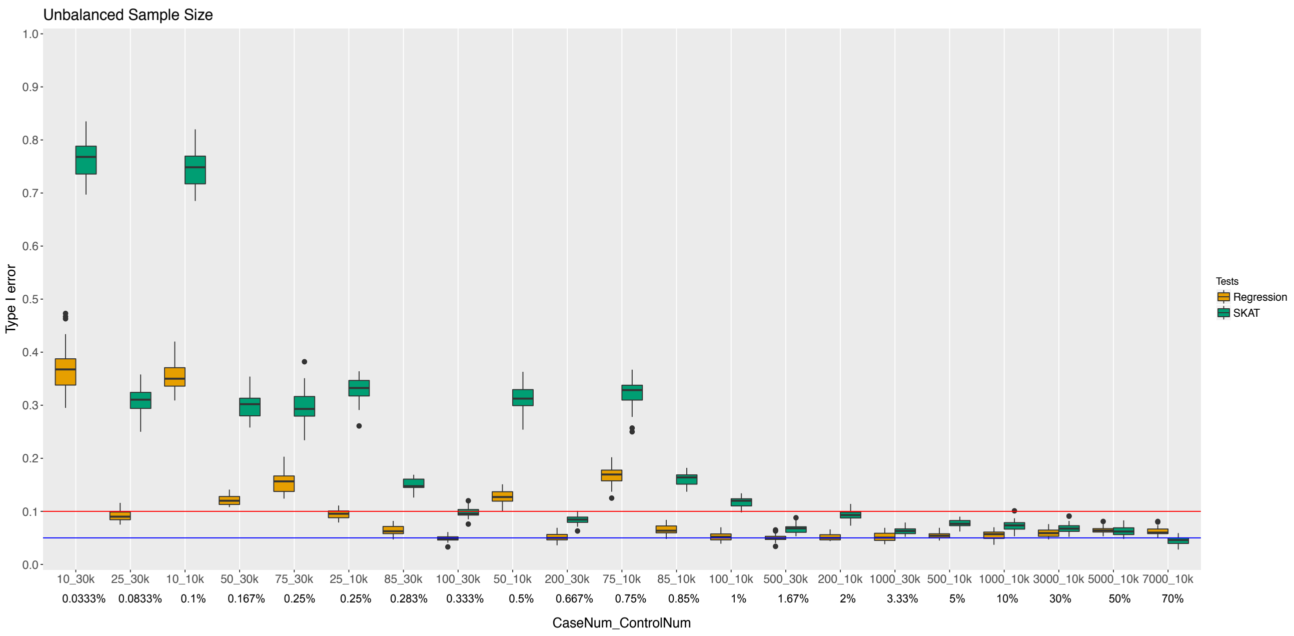

C

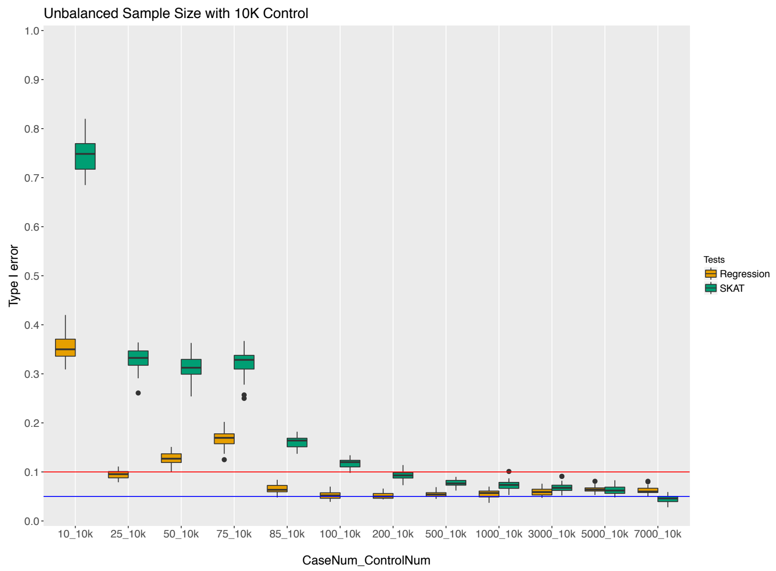

D

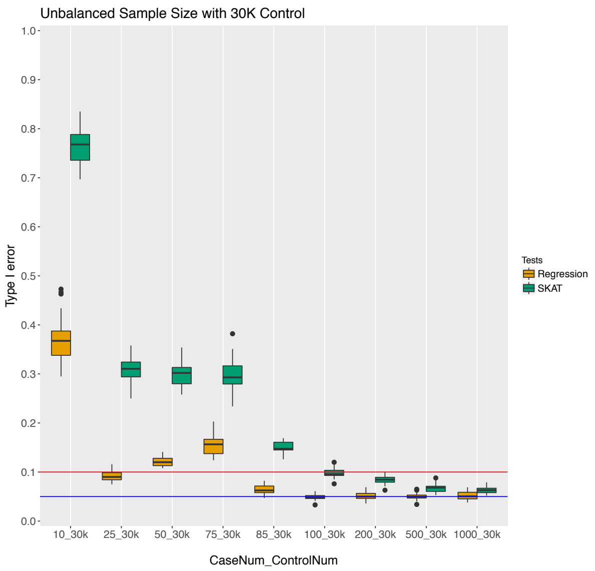

Figure S1 Type I error simulation results with MAF UB of 0.05. For visualization and comparison purposes, blue and red horizontal lines indicate type I error at 0.05 and 0.1 respectively. Figure (A) shows the results for type I error for an equal number of cases and controls for differing sample sizes. Note that the axis only goes to a type I error rate of 0.1. Figure (B) shows the type I error rate for different unbalanced cases and controls as arranged by case to control ratio. The axis is labeled by the number of cases then the number of controls for each simulation. The percentage of cases to controls is also listed below the number of cases and controls. Figures (C and D) show the results as ordered by the number of cases. Fig.1C has 10K control and Fig.1D has 30K control.

2. Power simulation results with with MAF UB of 0.05

A

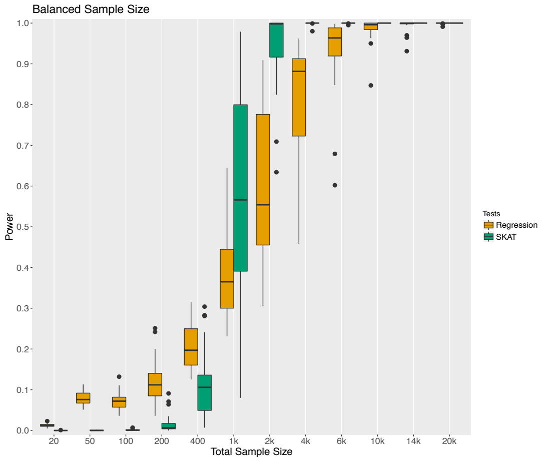

B

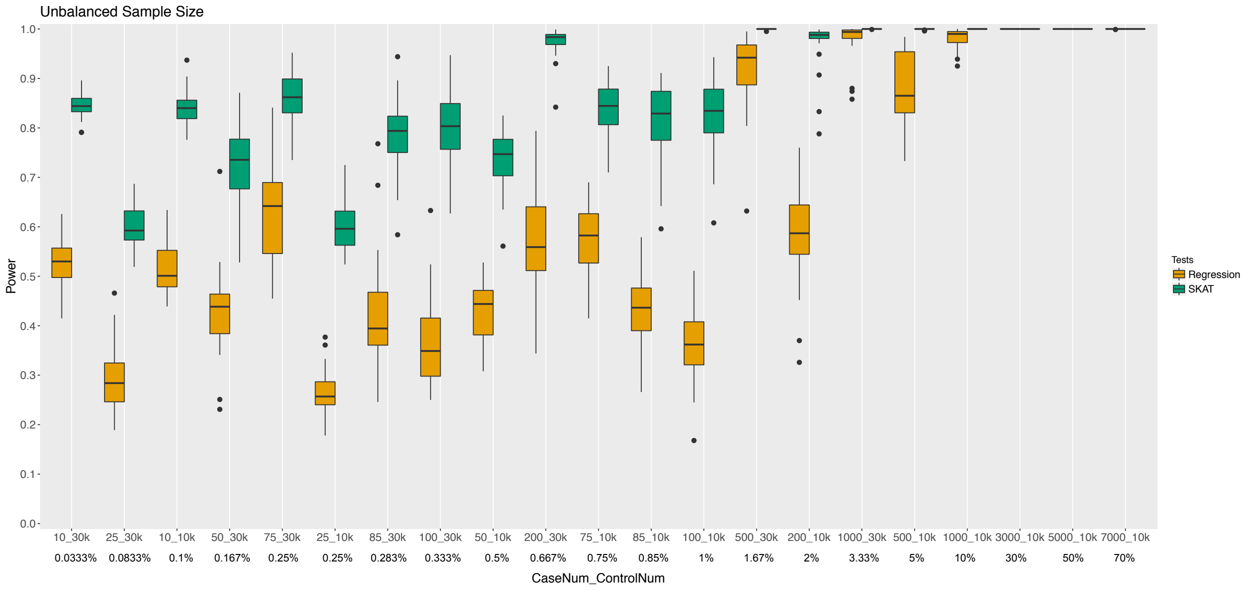

C

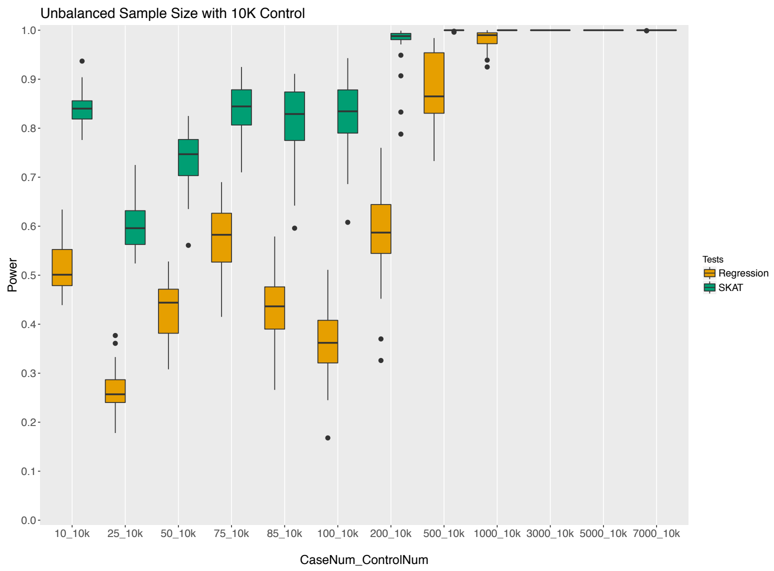

D

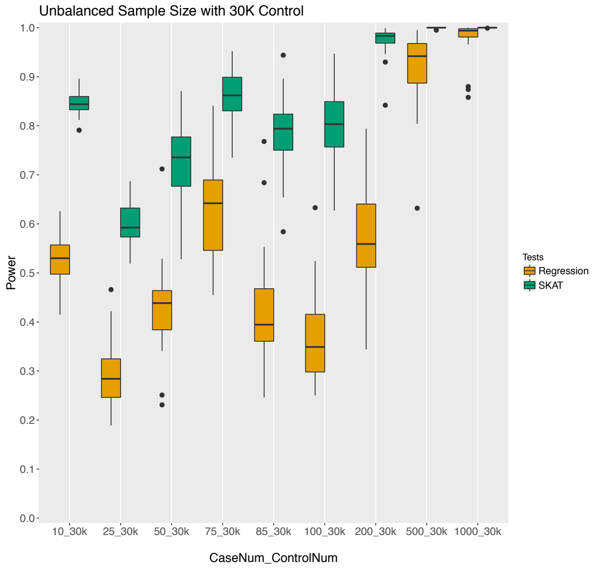

Figure S2 Power simulation results with cutoff for evaluated variation of MAF 0.05. Figure (A) shows the results when cases and controls are equal in number. Figure (B) shows the impact of unbalanced cases and controls on power ranked by the case/control ratio. The percent case to control ratio is listed below the x-axis. Figures (C and D) show the results for power with unbalanced cases and controls ordered by case number with 10K controls (C) and 30K controls (D).

3. Type I error and power simulation results using a constant ratio with MAF UB of 0.01

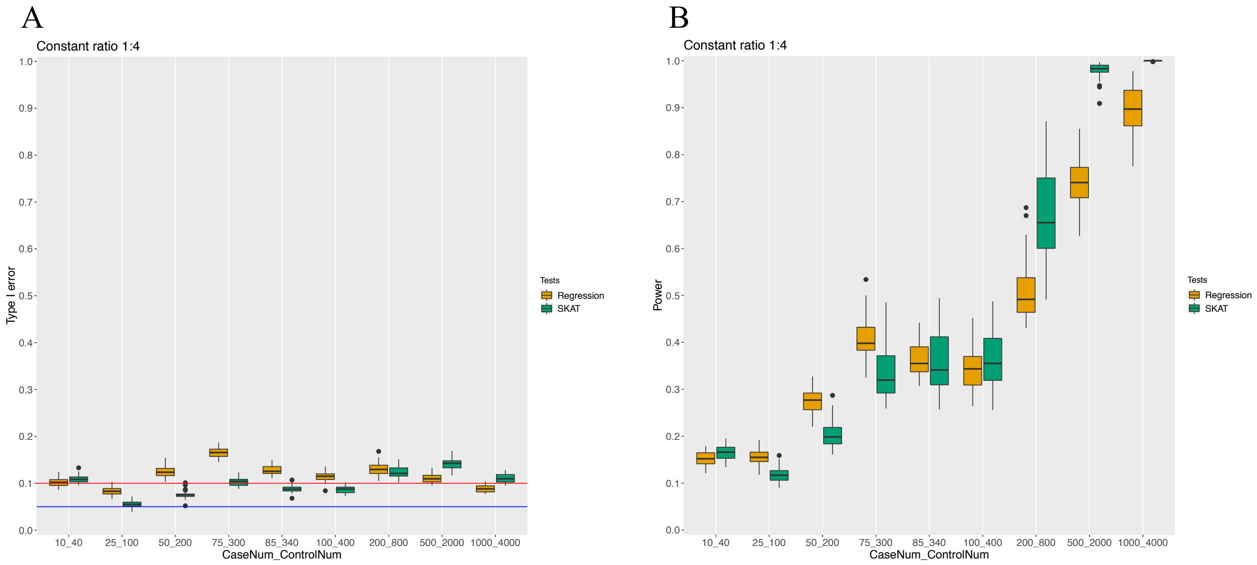

Figure S3 Type I error and power simulation results using a constant case to control ratio of 1:4 with MAF UB of 0.01. Figure (A) shows the results of type I error distribution. Blue and red horizontal lines indicate type I error at 0.05 and 0.1 respectively. Figure (B) shows the results of power distribution.

4. Type I error comparison when case control sample size is reversed

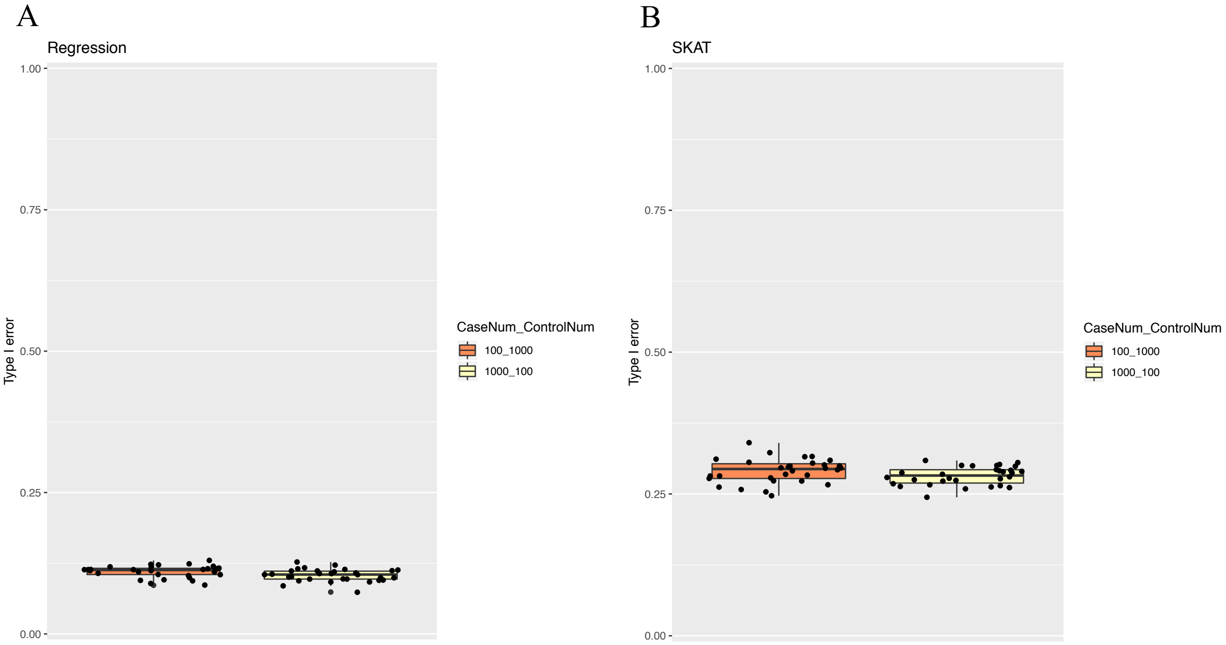

Figure S4 Type I error comparison of using 1. Case number of 100, control number of 1000 and 2. Case number of 1000 and control number of 100 for regression and SKAT. (A) shows the results for regression method. (B) shows the results for SKAT method.

5. Simulation results for case sample size of 200 and control sample size of 50k, 100k and 200k with MAF UB of 0.01

Table S1: Simulation design and results of larger controls (one replicate)

	Case Num	Control Num	Regression	SKAT
Type I error	200	50k	0.059	0.083
		100k	0.047	0.077
		200k	0.055	0.088
Power	200	50k	0.715	0.906
		100k	0.747	0.892
		200k	0.769	0.908
